# The role of primary care in informing and supporting people with limited health literacy in the Netherlands during the COVID-19 pandemic: a qualitative interview study

**DOI:** 10.1186/s12875-022-01723-w

**Published:** 2022-05-11

**Authors:** Bart Knottnerus, Monique Heijmans, Jany Rademakers

**Affiliations:** 1 Netherlands Institute for Health Services Research (Nivel), Utrecht, The Netherlands; 2 CAPHRI (Care and Public Health Research Institute), Department of Family Medicine, Maastricht University, Maastricht, The Netherlands

**Keywords:** COVID-19, Health literacy, Health communication, Primary health care, Professional-patient relations, Qualitative research

## Abstract

**Background:**

During the coronavirus disease 2019 (COVID-19) pandemic, people have been confronted with a large amount of information about the virus and the governmental measures against its spreading. However, more than a quarter of individuals have limited health literacy (HL), meaning that they have difficulty finding, understanding, and applying health information. The purpose of this interview study was to investigate how individuals with limited HL acquire information about COVID-19 and governmental measures, what difficulties they experience in understanding and applying it, and what may be needed to overcome these difficulties. We also addressed other problems that they might face as a result of the pandemic. Using our findings, we aimed to make recommendations on the possible role of primary care in informing and supporting patients with limited HL during the pandemic.

**Methods:**

Between June and October 2020, 28 individuals with limited HL were interviewed by phone (age range 20–84). The interviews were semi-structured and focused on the first months of the pandemic in the Netherlands (March/April/May 2020).

**Results:**

The participants generally found COVID-19-related information abundant and complicated, and sometimes contradictory. Information provision by their own health care professionals was highly appreciated, especially in the context of chronic illnesses. General health care problems resulting from COVID-19 measures were postponement of regular care and difficulty with digital contacts.

**Conclusions:**

Individuals with limited HL may benefit from provision of COVID-19-related information and support by their own health care providers. This applies in particular to patients with chronic illnesses. Primary care professionals are in the ideal position to take this role.

## Background

From the start of the coronavirus disease 2019 (COVID-19) pandemic, people have been confronted with an enormous amount of information, through television, newspapers, social media, and other social contacts. Moreover, this information is not always consistent and well-founded, and it changes over time [1, 2]. For tackling COVID-19, it is crucial that as many individuals as possible comply with measures taken by governments to prevent its spreading. This requires that people are able to select the right information, understand it and act upon it.

Many individuals have limited health literacy (HL), which is defined as “people’s knowledge, motivation and competences to access, understand, appraise, and apply health information in order to make judgments and take decisions in everyday life concerning healthcare, disease prevention and health promotion to maintain or improve quality of life during the life course” [3]. Population percentages of limited HL between 26 and 62% have been reported in the United States of America (USA) and different European countries [4–6]. And for less developed regions in the world, these percentages are likely to be even higher.

Previous research among individuals with limited HL has shown that information from governments and health care professionals is often too complex and not tailored to their information needs, levels of communication and preferred information channels [7, 8]. This may also be the case for information about COVID-19 [9]. As a possible intervention to improve information provision to individuals with limited HL, a bigger role for physicians has been proposed, since they may use their trust relationships to inform and support their patients [10].

So far, little research has been published on how individuals with limited HL cope with information about COVID-19 and what they need to improve their understanding of this information [11]. In three quantitative studies performed in March/April 2020, people with adequate HL were compared to people with limited HL. A study from the USA found that individuals with limited HL often had less worries about COVID-19 and possible infection with it [12]. Two other studies, from Australia and Germany, showed that people with limited HL had more difficulty finding and understanding information from the government [13, 14].

Although these studies provide some important evidence on the problems people with limited HL encounter, they do not give the in-depth insights needed to tailor information about COVID-19 to the specific needs of these people. Therefore, we performed a qualitative interview study. Our objectives were to investigate how individuals with limited HL acquire information about COVID-19 and governmental measures against it, what difficulties they experience in understanding and applying this information and what may be needed to overcome these difficulties. We also addressed other problems that individuals with limited HL might face as a result of the pandemic. Using our findings, we aimed to make recommendations on the possible role of primary care in informing and supporting patients with limited HL during the COVID-19 pandemic.

## Methods

### Methodological orientation

Since little research was available on COVID-19 information provision to individuals with limited HL, a qualitative, exploratory design was chosen. Semi-structured interviews were done, and analysed using a grounded theory approach [15].

### Participants

Individuals were eligible for study participation if they had insufficient or limited HL and were able to perform a one-hour phone interview in Dutch.

Participants were recruited from two national sources in the Netherlands: the National Panel of the Chronically ill and Disabled [16] and the Reading and Writing foundation [17]. Aiming at 30 interviews and taking into account that some eligible individuals would not be willing participate in our study, 34 individuals of varying age and with an equal male/female distribution were approached.

#### National Panel of the Chronically ill and Disabled

The National Panel of the Chronically ill and Disabled is a nationwide, longitudinal panel study in the Netherlands, established to gather information on the consequences of chronic illness and disability from a patient perspective. It is conducted by the Netherlands Institute for Health Services Research (Nivel) and consists of over 4000 individuals with a chronic illness or physical disability [16]. Upon entering the panel, these individuals have filled out a validated instrument that measures HL, the European health literacy survey (HLS-EU)-Q16 [18, 19]. This instrument shows whether an individual’s HL is insufficient, limited or adequate.

Panel members fill out questionnaires at home twice a year (in April and October). These questionnaires consist of fixed topics (e.g., quality of life, use of health services, experiences with healthcare and societal participation) and varying topics, which included experiences with the COVID-19 pandemic in April 2020. In this specific questionnaire, panel members were asked if they were also willing to be interviewed in depth about their experiences during the pandemic. From those who agreed and who had insufficient or limited HL (based on their HLS-EU-Q16 score), 22 individuals were invited by the panel coordinator participate in our study. Of these individuals, 19 were willing to participate and three refused because of health reasons.

#### Reading and Writing foundation

The Dutch Reading and Writing foundation is an organization that supports people who have difficulty with reading, writing, mathematics and digital skills [17]. The foundation approached 12 persons to participate in our study. All of these individuals experienced difficulties in reading and writing, implying that at least their functional HL was limited [20].

### Interviews

A semi-structured interview guide was developed, which was slightly adapted after the first four interviews, mainly by adding questions about testing and vaccination, as these topics became more relevant during the interview period. The first four interviews were not repeated or modified, since they provided sufficient valuable data (also on the topics that were added to the interview guide based on these interviews).

Each interview started with a short explanation of the study purpose and with asking permission to use anonymized quotes from the interview. Then, participants were told that the interview would focus on the first months of the COVID-19 pandemic in the Netherlands (March/April/May), but that they would also have the opportunity to talk about the current situation. Interview topics were: experiences with COVID-19 and its consequences (e.g., personal problems, general worries, social contacts, financial issues), sources of information about COVID-19 and governmental measures, experienced quality of information, compliance with measures, preferred sources of information and need for support.

All interviews were conducted by phone by either the first or the second author. Writing the interview guide, conducting the interviews, and coding the data were all performed in Dutch.

The interviews took place between June and October 2020, a period in which the COVID-19 measures from the Dutch government were relatively mild until they were tightened up from the second half of September.

### Analysis

The interviews were audio-recorded and transcribed. All information that might have been traceable to participants was omitted. Each transcript was coded by the researcher that did the corresponding interview, using MAXQDA 11. Coding was done according to the predefined interview topics. Extra codes were added if information did not fit well into these topics or needed more specific coding. All transcripts and codes were discussed in the research team to reach consensus.

## Results

Of the 31 individuals that were contacted, three did not answer their phone despite several attempts. Since data saturation had been reached, no additional participants were approached. Thus, 28 individuals were interviewed; 13 were male and 15 female; their age ranged from 20 to 84 (Table 1). All participants had at least one health problem, in almost all cases being a chronic illness. All interviews lasted an hour or less.

**Table 1:** Overview of interview participants

Sex	Age	Self-reported health problems	Month of interview (in 2020)
Male	74	Stroke Fatigue	June
Male	49	Severe asthma Obesity Somberness	June
Female	54	Lung disease	June
Female	20	Metabolic disease Acquired brain impairment Fear symptoms	June
Female	31	Fibromyalgia Muscle disease Shoulder symptoms Obesity	August
Female	84	Pneumonia Chronic cold	September
Female	48	Severe asthma Thrombosis	September
Female	68	Heart problems Diabetes	September
Male	66	COPD Heart problems Crohn’s disease	September
Male	68	Diabetes	September
Male	64	COPD	September
Male	61	COPD	September
Male	75	Chronic leukaemia	September
Male	77	Sarcoidosis	September
Female	47	Sarcoidosis	September
Female	64	Fear symptoms	September
Male	73	Lung disease	September
Male	82	Arthrosis Bypass	September
Female	55	Muscle dystrophia	September
Female	58	Several strokes Thrombosis	September
*Female*	*54*	*Diabetes* *Bowel disease*	*September*
*Male*	*65*	*Shoulder symptoms* *Diabetes* *Obesity*	*September*
*Female*	*60*	*Asthma* *Often pneumonia*	*October*
*Male*	*64*	*Back symptoms* *Obesity*	*October*
*Male*	*68*	*Heart problems*	*October*
*Female*	*79*	*Rheumatoid arthritis*	*October*
*Female*	*30*	*Mental symptoms*	*October*
*Female*	*70*	*Chronic bowel disease* *Asthma*	*October*

Participants in italics were interviewed during a period in which governmental measures were tightened up in the Netherlands

### Information about COVID-19 and governmental measures

All participants experienced an extensive amount of general information about COVID-19 and the governmental measures against it, and for many it was too abundant. They mainly acquired the information by following press conferences given by the government on television, watching talk shows that tried to clarify the information from these conferences, looking for additional information on the internet, and talking with family, friends, and colleagues. From all this information, a few participants were able to select what was relevant to them, but most were not and felt overwhelmed or scared by the abundance. Moreover, the language used by the government was too difficult for many participants. Besides, information from the government was often experienced as contradictory, especially with respect to specific measures additional to the basic ones (keeping distance, washing hands). Contradictory information also came from the many experts on television, whose different opinions often led to confusion. For several participants, the experienced abundance, complexity, and inconsistency of the received information made them stop following it.

*“Then you read it again, or I call my sister to ask what they mean. I ask my children. Or I think ‘forget it’, because it’s all too difficult.” (Female, 54).*

*“Because they don’t agree. One says this and another one says that.” (Male, 65).*

A few participants were informed about COVID-19 by their own health care professionals, which they highly appreciated. They considered it more reliable and of more personal relevance than other information. Moreover, some other participants explicitly mentioned having missed their health care professional advising them on how to cope with the pandemic, especially in the context of their chronic illnesses.

### Need for answers to additional questions

Many participants had questions about COVID-19 that were not satisfactorily answered through general information. These mainly consisted of personal questions about health-related consequences and general questions about the origin and characteristics of the virus, the differences with influenza, and policy choices. Missing clear explanations sometimes led to participants developing their own theories and even questioning whether the virus really existed. These doubts negatively influenced compliance with governmental measures.

A large category of unclarities concerned testing for COVID-19, which often made participants hesitant to undergo a test. These unclarities included doubts about the test reliability and perceptions that the test would cost them money or that the procedure might damage something in the body. A substantial number of participants mentioned that a test result was only a snapshot and therefore doubted its usefulness. Moreover, some were reassured by a previous negative test result when they got symptoms again.

*“The testing is all very well, but I can be negative today and get corona next week. It’s not a guarantee.” (Male, 61).*

*“Would you test again? Every time again? Not me. I had no corona back then and I have the same now.” (Female, 47).*

At the time of our interviews, all COVID-19-vaccines were still under development. Lack of knowledge made several participants resistant to be vaccinated when that would be possible. For instance, participants said that vaccination would only make sense if symptoms were present, and one-on-one comparisons with influenza vaccination were made.

*“As long as I’m not ill I don’t need it.” (Female, 68).*

*“I definitely won’t vaccinate, I don’t do that for the flu either. I can tell you something about that: I haven’t been vaccinated for six years and I haven’t been ill since then.” (Male, 49).*

### Trust in information providers

Trust in the government was important for understanding information and complying with measures. This concerned both general trust and trust in the specific context of COVID-19. Distrust was often stimulated by the belief that measures were driven by political or financial interests and by discrepancies between received information and personal experiences.

*“I watch the news, but I don’t recognize it. I don’t see anything here. No people with face masks. And I don’t know anyone who’s ill.” (Female, 55).*

Trust in health care professionals was generally higher but was lacking for some participants who had negative experiences related to other health issues. These participants were less tended to accept information and support from their health care professionals.

*“I say I feel it’s becoming an angina. No, no, it’s just a cold. Okay. And then I come back a week later, and then I have a mega heavy throat infection, because she didn’t listen. So yes, if you have that with corona, I’m afraid they just won’t listen to me.” (Female, 31).*

### Postponement of health care

Several participants had experienced postponement of care for existing health problems. This concerned especially hospital care, physiotherapy, and mental care. The postponement often led to deterioration of the specific health problem or to mental symptoms.

*“In the beginning of March, I was in the hospital for all kinds of investigations. And those investigations were very important, because without them my treatment couldn’t go on. But they were all stopped at once of course. Yes, that was very bad … at that moment I was at a low point in my life.” (Female, 20).*

### Accessibility of health care

Many contacts with health care professionals became digital, which was difficult for some participants. They needed help with the use of the internet or got frustrated by the lack of personal contact with a health care professional. A few participants were unpleasantly surprised by the resistance they felt from their primary care professionals when they called them to get a physical consultation because of possible COVID-19 symptoms.

*“I don’t do internet appointments because writing is a big problem for me. Video calls? Skype? No, I’ve never done that. I don’t even know if it’s possible.” (Female, 47).*

*“And then they said: ‘Yes, you can breathe because you are talking’. But because I just really, I just couldn’t do anything. I wanted to go there. Then they said: ‘We can’t do anything. You just have to stay in quarantine and do nothing. Those are the rules. Period.’” (Female, 30).*

## Discussion

### Main findings

For our study participants, general information about COVID-19 and governmental measures was often too difficult. The information was considered abundant, complicated, and sometimes contradictory. Participants valued personalized information from their own health care professionals, especially in the context of chronic illnesses. However, only a few had received such information. Trust in information providers was crucial for understanding information and complying with measures. Whereas some participants had little trust in the government, a large majority trusted their health care professionals. General health care problems resulting from COVID-19 measures were postponement of regular care and difficulty with digital contacts.

### Role of primary care

Based on our interview results, we have identified three themes where primary care professionals could be of importance to their patients with limited HL: (1) as a proactive source of personalized information about COVID-19; (2) to answer individual questions about COVID-19, the governmental measures against it and the considerations behind these measures; and (3) to offer advice and support in case of deterioration of other health issues due to COVID-19 measures. For all three themes, two crucial conditions came forward: accessibility and trust. With respect to accessibility, having difficulty with digital consultations was specifically mentioned. With respect to trust, a lack of faith in health care professionals was a barrier to accepting information and support from them.

#### Providing information about COVID-19

Several participants lacked basic knowledge about the virus, the background of governmental measures, and the use of testing and vaccinating, which revealed a need for tailored information provision. Personal, proactive information provision by a health care professional was highly appreciated, especially in the context of a chronic illness. Knowing the context of their patients, primary care professionals are in the ideal position to take this role.

#### Answering individual questions about COVID-19

Many participants had questions about COVID-19 that were not satisfactorily answered through general information, about both general and personal issues (e.g., policy choices and own health). Lacking other personal information channels tailored to individuals with limited HL, they may highly benefit from discussing these questions with their own primary care professionals.

#### Offering support for other health issues

Deterioration of other health issues and mental symptoms due to postponement of specialized care (especially hospital care, physiotherapy and mental care) was frequently mentioned as a consequence of COVID-19, implying a need for support. Primary care professionals would be the most suitable providers of this support.

Our findings are supported by several other studies. The need to tailor COVID-19-related information to specific groups and the possible role for primary care in that were emphasized before [21, 22]. For individuals with limited HL specifically, being approached by a health care professional was mentioned as an effective way to be informed about COVID-19 [23]. This has been considered even more important when trust in the government is lacking [10]. Primary care professionals can take this role, provided that they visibly demonstrate the trust needed [24]. Also, with respect to general health issues, primary care has been reported to be an important information provider for individuals with limited HL, especially among the elderly and chronically ill [25]. However, as was reported by an interview study among primary care professionals in the USA [26], these individuals are the most difficult to reach and manage during a pandemic (e.g., due to difficulty with digital consultations), which stresses the needed attention for accessibility we described. In line with our findings, it has been recommended to prioritize and proactively contact vulnerable patients in primary care during a pandemic [27].

### Strengths and limitations

To our knowledge, this is the first study that reports qualitative, in-depth results on the ways in which individuals with limited HL cope with information about COVID-19 and the governmental measures against it.

To select people with limited HL, we used a valid and reliable self-report questionnaire that addresses difficulties with accessing, understanding, appraising and applying information, the HLS-EU-Q16 [18, 19]. A self-report questionnaire implies that a certain level of literacy is needed, thereby excluding individuals who have severe difficulties with reading and writing. However, the HLS-EU has been shown suitable to be used for people with limited literacy [28]. To include people with reading and writing difficulties, we purposively sampled this group through the Reading and Writing Foundation; although HL was not formally assessed in these individuals, their difficulties in reading and writing implied that at least their functional HL was limited [20].

It might be argued whether our findings are specific for people with limited HL, as they may apply to a larger part of the general population. However, difficulty with understanding and applying information is more common among people with limited HL, and so is a lack of trust in the government [10]. Also, in practice, primary care professionals do not need to only approach patients with limited HL when informing about COVID-19, since other patients value clear information as well.

All our participants had at least one health problem, which makes our results in particular applicable to individuals with chronic illnesses or other health-related issues. However, these are exactly the individuals who benefit most from information provision by primary care professionals [25].

## Conclusions

For individuals with limited HL, general information about COVID-19 and governmental measures against its spreading can be overwhelming, confusing, and hard to apply. In line with previous literature, our findings show that primary care professionals can have an important role as personal providers of COVID-19-related information and support for individuals with limited HL. This applies in particular to patients with chronic illnesses, who may have specific questions and needs concerning other health issues in the context of COVID-19.

## Data Availability

The data analysed during the current study are available from the corresponding author on reasonable request.

## Abbreviations

COVID-19: Coronavirus disease 2019
HL: Health literacy
USA: United States of America
HLS-EU: European health literacy survey
